# Rationale on the High Radical Scavenging Capacity of Betalains

**DOI:** 10.3390/antiox8070222

**Published:** 2019-07-13

**Authors:** Karina K. Nakashima, Erick L. Bastos

**Affiliations:** Departamento de Química Fundamental, Instituto de Química, Universidade de São Paulo, São Paulo, SP 05508-000, Brazil

**Keywords:** betalain, antioxidant, radical scavenger, natural pigments, redox mediator

## Abstract

Betalains are water-soluble natural pigments of increasing importance as antioxidants for pharmaceutical use. Although non-phenolic betalains have lower capacity to scavenge radicals compared to their phenolic analogues, both classes perform well as antioxidants and anti-inflammatory agents *in vivo*. Here we show that *meta*-hydroxyphenyl betalain (*m*-OH-pBeet) and phenylbetalain (pBeet) show higher radical scavenging capacity compared to their *N*-methyl iminium analogues, in which proton-coupled electron transfer (PCET) from the imine nitrogen atom is precluded. The 1,7-diazaheptamethinium system was found to be essential for the high radical scavenging capacity of betalains and concerted PCET is the most thermodynamically favorable pathway for their one-electron oxidation. The results provide useful insights for the design of nature-derived redox mediators based on the betalain scaffold.

## 1. Introduction

Oxidants play a major role in metabolism [1,2,3]. Despite their importance in several biological processes, such as cell signaling, proliferation and differentiation, the overproduction of oxidants has been linked to harmful health effects [4]. The interpretation of scientific data for the action of oxygen, nitrogen and sulfur oxidants *in vivo* has changed over the decades. For example, cell death caused by oxidative stress is now considered—in certain cases—as an oxidant-triggered physiological process required to maintain physiological homeostasis rather than the root cause of all evil in biological systems [5].

Antioxidants have been considered to be must-use food supplements against the deleterious effects of oxidants [6,7]. This idea was broadly advertised in the second-half of the 20th century and echoes in the 21st century [8,9]. Natural products having high antioxidant capacity (thermodynamics, amount of radicals scavenged) and/or activity (kinetics, reactivity towards radicals) are high added value substances. However, several natural antioxidants can act as pro-oxidants, depending on the dose. Vitamin C and flavonoids, such as epicatechin gallate (ECG) from green tea, are examples of widely used antioxidants that show pro-oxidant action under certain conditions [10,11].

Betalains are chiral water-soluble natural products that replace anthocyanins in the pigmentation of most families of Caryophyllales plants [12,13,14,15]. Betanin (betanidin 5-*O*-glucoside) is a phenolic betalain found in beetroots (*Beta vulgaris* L.) that has been used as an U.S. Food and Drug Administration-approved food colorant additive [16]. The chemical versatility of betanin has led to its application as a redox mediator in dye-sensitized solar cells [17] and hydrogen production systems [18,19], for the synthesis of metal nanoparticles [20], as an anti-inflammatory agent [21], and as an antioxidant [22]. The antioxidant capacity of betanin is comparable to that of ECG due to the presence of a 5-*O*-glucosyl catechol moiety and a 1,7-diazaheptamethinium [23] system [24,25,26]. We have found that *N*-(3-hydroxylphenyl)betalain (*m*-OH-pBeet) is an adequate model to study the mechanism of antioxidant action of betanin because they share the same radical scavenging capacity [27]. Although the antioxidant action of *m*-OH-pBeet was rationalized in terms of proton-coupled electron transfer (PCET), the importance of the 1,7-diazaheptamethinium system for the antioxidant potential of betalains could not be elucidated [27]. 

Here we discriminate between the contributions of the phenolic moiety and the 1,7-diazaheptamethinium system on the antiradical properties of betalains and provide evidence that betalains undergo concerted PCET, transferring electrons and protons that are not necessarily at the same reaction site. The radical scavenging capacity, electrode potentials and electronic properties of phenyl betalain (pBeet) and *m*-OH-pBeet were compared to those of their corresponding *N*-methyl analogues, *N*-methyl-*N*-phenylbetalain (mepBeet) and *N*-methyl-*N*-(3-hydroxyphenyl)betalain (*m*-OH-mepBeet), in which PCET involving the imino group is precluded. The presence of the *N*-methyl group attached to the imine nitrogen atom changes the charge delocalization profile of betalains, lowers the radical scavenging capacity, and increases the hydrolytic stability compared to the non-methylated analogues. 

## 2. Materials and Methods 

### 2.1. General

All chemicals were purchased from Sigma-Aldrich (Milwaukee, USA) and used without further purification, except as otherwise stated. Solutions were prepared using deionized water (18.2 MΩ cm at 25 °C, total organic carbon ≤ 4 ppb, Milli-Q, Millipore (Massachusetts, USA). Values are expressed as mean ± standard deviation of three completely independent replicates.

### 2.2. Semisynthesis of Betalains

pBeet, mepBeet, *m*-OH-pBeet, and *m*-OH-mepBeet were semisynthesized using betalamic acid according to a procedure adapted from Schliemann and coauthors [28]. In a 5-mL one-necked round-bottom flask protected from light were placed a solution of betalamic acid (1 mmol L^−1^) in ethyl acetate (2 mL) and solid aminophenol (10 equivalents). The suspension was submitted to ultrasonic irradiation (40 kHz, bath) until the solid had dissolved, kept at 25 °C for 30 min, and the resulting solution was kept in a freezer (−20 ± 2 °C) overnight. The resulting suspension was centrifuged (5000 ×*g*, 5 °C, 5 min) and the precipitate was washed twice with ethyl acetate. Products were purified through semi-preparative reversed phase HPLC/PDA (Phenomenex Luna C18 25 cm × 1 cm, 5 μm, isocratic 20% B; solvent systems, A: formic acid (0.1% *v/v*) in water, B: formic acid (0.1% *v/v*) in MeCN/water (90/10 *v/v*); flow rate: 3 mL min^−1^; monitoring at 254 nm, 400 nm, and 500 nm). Fractions containing betalains were combined, frozen and lyophilized. Products were kept in the dark at −20 °C and submitted to UV-vis spectrophotometric analysis immediately before use. The average yield is 20%.

**pBeet**; ^1^H NMR (500 MHz, Methanol-*d*_4_) δ 8.50 (d, *J* = 12.0 Hz, 1H), 7.43 (t, *J* = 7.7 Hz, 2H), 7.37 (d, *J* = 8.0 Hz, 2H), 7.25–7.19 (m, 1H), 6.36 (s, 1H), 6.21 (d, *J* = 12.0 Hz, 1H), 4.52 (t, *J* = 7.4 Hz, 1H), 3.43 (dd, *J* = 17.5, 7.4 Hz, 1H), 3.24 (dd, *J* = 17.5, 7.4 Hz, 1H). HPLC-ESI(+)/MS: R_t_ = 6.2 min (see *SI*), *m/z* 287.1. Magenta solid.

***m*-OH-pBeet**; ^1^H NMR (500 MHz, Methanol-*d*_4_) δ 8.35 (bs, 1H), 7.21 (t, *J* = 8.1 Hz, 1H), 6.80 (dd, *J* = 8.1, 2.3 Hz, 1H), 6.75 (t, *J* = 2.3 Hz, 1H), 6.64 (dd, *J* = 8.1, 2.3 Hz, 1H), 6.35 (s, 1H), 6.15 (d, *J* = 12.7 Hz, 1H), 4.44 (t, *J* = 8.0 Hz, 1H), 3.24 (dd, *J* = 17.9, 8.0 Hz, 2H). HPLC-ESI(+)/MS: R_t_ = 4.7 min (see *SI*), *m/z* 303,1. Magenta solid.

**mepBeet**; ^1^H NMR (500 MHz, Methanol-*d*_4_) δ 8.18 (bs, 1H), 7.51 (t, *J* = 8.0 Hz, 2H), 7.42 (d, *J* = 8.0 Hz, 2H), 7.38–7.32 (m, 1H), 6.49 (s, 1H), 6.14 (bs, 1H), 4.45 (t, *J* = 7.8 Hz, 1H), 3.61 (s, 3H), 3.20 (dd, *J* = 17.4, 7.8 Hz, 1H). HPLC-ESI(+)/MS: R_t_ = 6.7 min (see *SI*), *m/z* 301,2. Red solid.

***m*-OH-mepBeet**; ^1^H NMR (500 MHz, Methanol-*d*_4_) δ 8.15 (bs, 1H), 7.29 (t, *J* = 8.1 Hz, 1H), 6.91–6.72 (m, 3H), 6.48 (s, 1H), 6.13 (bs, 1H), 4.46 (t, *J* = 7.6 Hz, 1H), 3.57 (s, 3H), 3.18 (dd, *J* = 17.4, 7.6 Hz, 1H). HPLC-ESI(+)/MS: R_t_ = 5.4 min (see *SI*), *m/z* 317,2. Red solid.

### 2.3. Radical Scavenging Capacity

The Trolox Equivalent Antioxidant Capacity (TEAC) assay was used to determine the antiradical capacity of the betalains [29]. ABTS (2,2’-azinobis-(3-ethyl-benzothiazoline-6-sulfonic acid), 7 mmol L^−1^) was partially oxidized by potassium persulfate (2.45 mmol L^−1^) producing a solution of ABTS^+•^/ABTS in water. The reaction was carried out in the dark at 25 °C for 16 h. The stock solution of ABTS^+•^/ABTS was diluted to an absorbance of 0.7 (46.7 μmol L^−1^ ABTS^+•^) at 734 nm using Britton-Robinson buffer (acetate/phosphate/borate, 0.4 mol L^−1^, at the different pHs). After addition of antioxidant (20–60 μL, final concentration within the μmol L^−1^ range), changes in the absorbance at 734 nm were monitored for a period of 6 to 120 min (ΔA). The antioxidant capacity is proportional to the slope of the linear correlation between ΔA and the antioxidant concentration, α. The TEAC is the α_sample_/α_Trolox_ ratio.

### 2.4. Computational Methods

The theoretical study of PCET was carried out using the Density Functional Theory (DFT)-optimized geometries of pBeet, mepBeet, *m*-OH-pBeet, and *m*-OH-mepBeet, their deprotonated forms (at N1–H, N9–H and PhO–H, when feasible), and the corresponding radical cations. All equilibrium geometries were optimized at the SMD(water)/M06-2X/6-311++G(d,p) level [30,31,32], and stationary points were characterized as minima based on vibrational analysis. The reported energies include the zero-point energy and thermal corrections (T = 298.15 K) to electronic energies. Gaussian09 rev. D.01 program suite was used for all calculations [33]. Equations (1)–(5) were used to calculate the thermodynamic parameters governing the radical scavenging mechanism [34].

(Bond dissociation enthalphy, BDE, BetH) = *H*(Bet^•^) + *H*(H^•^) – *H*(BetH) (1)

(Ionization potential, IP, BetH) = *H*(BetH^+•^) + *H*(e^−^) – *H*(BetH) (2)

(Proton dissociation enthalpy, PDE, BetH^+•^) = *H*(Bet^•^) + *H*(H^+^) – *H*(BetH^+•^) (3)

(Proton affinity, PA, BetH) = *H*(Bet^−^) + *H*(H^+^) – *H*(BetH) (4)

(Electron transfer enthalpy, ETE, Bet^−^) = *H*(Bet^•^) + *H*(e^−^) – *H*(Bet^−^) (5)

The gas phase enthalpies of the proton (6.197 kJ mol^−1^), electron (3.146 kJ mol^−1^) and hydrogen atom (–1306 kJ mol^−1^), as well as the solvation enthalpies (water) of the proton (–1055.7 kJ mol^−1^), electron (–77.5 kJ mol^−1^), and hydrogen atom (–4 kJ mol^−1^) were taken from the literature [35,36]. Phenol and the phenoxyl radical were used as reference compounds to calculate the ΔBDE^PhOH^ (Equation (6)) [37].

ΔBDE^PhOH^ = *H*(Bet^•^) + *H*(PhOH) – *H*(BetH) – *H*(PhO^•^) (6)

The theoretical BDE of phenol at the SMD/M062X/6-311++G(d,p) level is 363.3 kJ mol^−1^ (86.8 kcal mol^−1^), which is in general agreement with the literature values of 86.7 kcal mol^−1^, 87.7 kcal mol^−1^, 88.0 ± 1 kcal mol^−1^, and 88.7 kcal mol^−1^ [38]. The values of BDE and bond dissociation free energy (BDFE) are expected to be similar since entropic changes are usually small for all-organic PCET reactions [38].

## 3. Results and Discussion

### 3.1. Semisynthesis and Electronic Properties of pBeets and mepBeets

**Figure 1:**
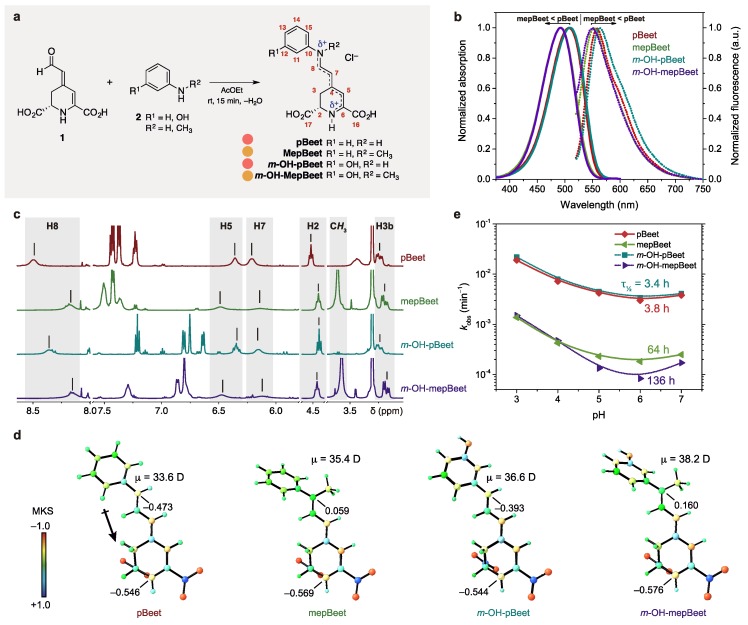
Semisynthesis and characterization of pBeets and mepBeets. (**a**) Acid-catalyzed coupling of betalamic acid (1) and anilines 2 in ethyl acetate. Atom numbering is shown in red and the colored circles indicate the color of each compound. (**b**) Normalized absorption (solid line) and fluorescence (dotted line) spectra of pBeets and mepBeets in BR buffer pH 6; excitation at 490 nm. (**c**) ^1^H NMR spectra of pBeets and mepBeets (70 μmol L^−1^, 500 MHz, CD_3_OD at 293 K). (**d**) Geometries of pBeets and mepBeets optimized at the SMD(water)/M06-2X/6-311++G(d,p) level and partial charges of the nitrogen atoms according to the Merz–Kollman–Singh (MKS) scheme constrained to reproduce the dipole moment (μ). The p*K*_a_s of the carboxyl groups of betanin and indicaxanthin are ca. 3.5 [50,51] and, therefore, we show the carboxy-deprotonated forms of these model betalains. (**e**) Effect of pH on the observed rate constant (*k*_obs_) and half-lives for the hydrolysis of the pBeets and the mepBeets at pH 6, 25 °C.

Four model betalains were semisynthesized from betalamic acid (1) (extracted from hydrolyzed beetroot juice) and the anilines 2 using a scaled up procedure based on the method of Schliemann and coauthors [28] (Figure 1a). Ethyl acetate was used as the organic solvent [39] for both the extraction of 1 and the semisynthesis. The HPLC-DAD-MS/ESI(+) analysis and NMR spectra are presented in Figures S1–S8. 

The absorption and fluorescence maximum wavelengths of the *N*-methyl betalains (mepBeets) in water are blue-shifted compared to those of pBeet and *m*-OH-pBeet (Table 1, Figure 1b). Consequently, mepBeets are orange (color data, CIE L 86 a –4 b 58), while the non-methylated analogues are bright salmon (CIE L 78 a 35 b 8). The color of pBeet was first described by Mabry and coauthors in the early 1970s when they performed seminal experiments coupling betalamic acid with amines and amino acids, and later by Gandía-Herrero and collaborators [40,41]. The fluorescence quantum yields (Φ_FL_) of mepBeets are ca. 30% lower than those of pBeet and *m*-OH-pBeet (Table 1), in agreement with reports on the effect of the presence of electron-donating substituents in the imine portion of aryl betalains [41,42,43,44]. Although pBeet is more fluorescent than the other three derivatives, i.e. 35% more fluorescent than *m*-OH-pBeet, it is still less emissive in solution than the natural betalain vulgaxanthin II (Φ_FL_ in water = 7.3 × 10^−3^) [45]. 

^1^H NMR data show that the *N*-methyl group shields H8 and deshields H5 of these model betalains, possibly due to positive hyperconjugation (σ^2^_CH_ → *p*_N_) [46] and because the aromatic ring and the 1,7-diazaheptamethinium group of the mepBeets are not coplanar (Figure 1c,d). The mepBeets show higher dipole moments (μ) compared to the pBeets, as evidenced by the increase of the positive charge density at N9 and decrease at N1 (Figure 1d). The greater charge localization of mepBeets explains their blue-shifted absorption spectra compared to pBeet and *m*-OH-pBeet, as well as the observed changes in the chemical shifts of H8 and H5. 

*N*-Methyl betalains are more persistent in aqueous solution compared to pBeet and *m*-OH-pBeet. The pH dependence of the observed rate constant for hydrolysis (*k*_obs_) and the corresponding half-lives show the inverted bell-shaped profile typical of betalains (Figure 1e) [16]. Although all betalains show maximum persistence at pH 6, the half-lives of mepBeets are roughly 25-times higher than those of pBeet and *m*-OH-pBeet at 25 °C, i.e., 100 h vs. ca. 4 h. The imine *sp*^2^ carbons of non-methyl betalains are more activated towards nucleophilic attack by water and, consequently, hydrolyze faster than imino-protonated mepBeet and *m*-OH-mepBeet. This result is important for the design of betalains that are less sensitive to hydrolysis, thus broadening the application of this class of compounds [47,48,49]. 

### 3.2. Radical Scavenging Capacity

**Figure 2:**
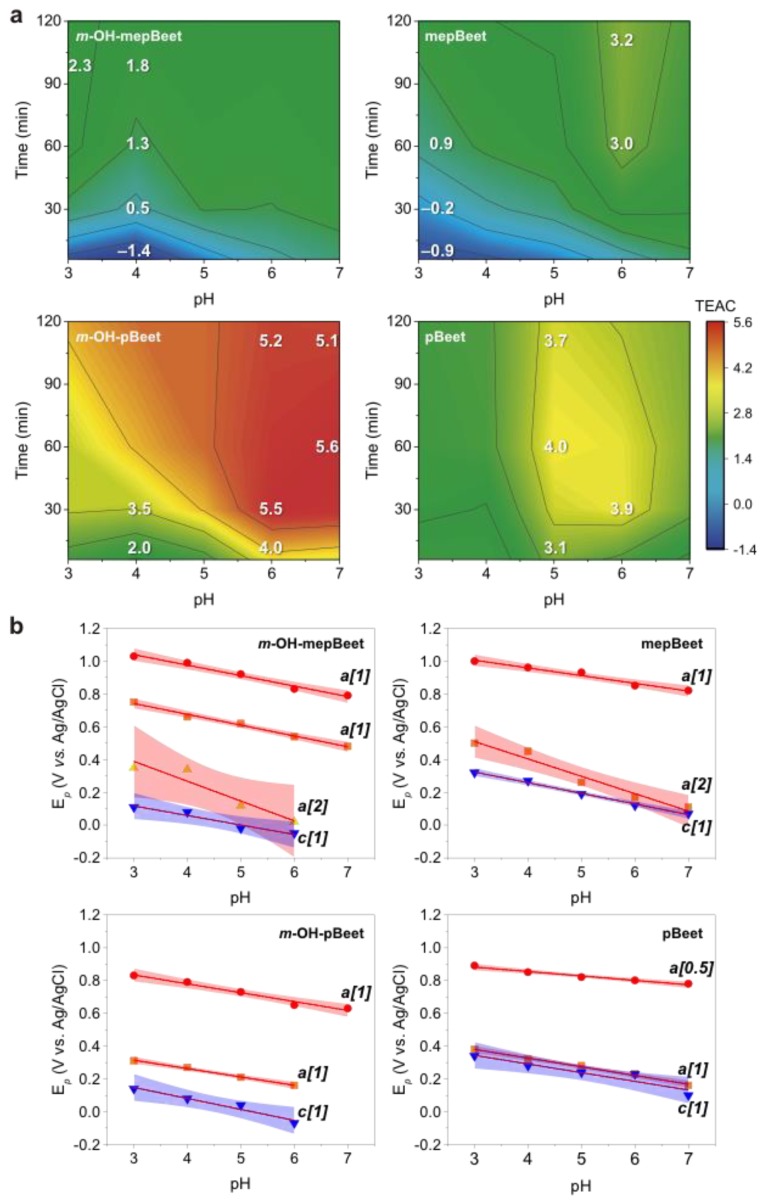
Antiradical capacity and redox properties of pBeet, mepBeet, *m*-OH-pBeet and *m*-OH-mepBeet. (**a**) Colored contour map of Trolox equivalent antioxidant capacity (TEAC) of the betalains as a function of the pH (*x*-axis) and reaction time (*y*-axis). Red to blue color gradient indicating high to low (5.6 to −1.4) TEAC; selected TEAC contour values are indicated on the surface for clarity. Raw data is presented in Table S1. Reaction conditions: Britton–Robinson (BR) buffer (40 mmol L^−1^), [ABTS^+•^] = 46.7 μmol L^−1^, [Trolox]: 0.5–4 μmol L^−1^, [betalain]: 0.3–1.5 μmol L^−1^, at 25 ± 1 °C. (**b**) Dependence of the electrode potential (E*_p_*) of the betalains on the pH (BR buffer). Straight lines show linear regressions (confidence interval of 95%, red for anodic and blue for cathodic peaks); numbers in brackets are the proton to electron ratio. Cyclic voltammograms are presented in Figure S10.

The radical scavenging capacity of pBeets and mepBeets was determined using the Trolox Equivalent Antioxidant Capacity (TEAC)/ABTS^+•^ colorimetric assay [29]. In this assay, the reduction of the green-colored ABTS^+•^ by antioxidants is quantified over a period of six minutes and the change in absorbance at 734 nm is used to calculate the TEAC value (see Methods). Since the reaction of ABTS^+•^ with less reactive antioxidants may take more than 6 min to reach equilibrium [53], we monitored the reaction for up to 2 h at pH ranging from 3 to 7. Results are presented as colored contour maps in Figure 2a.

After 6 min in the presence of mepBeet or *m*-OH-mepBeet under acidic conditions (pH range 3–5), the concentration of ABTS^+•^ increased, resulting in negative TEAC values and implying pro-oxidant action of *N*-methyl betalains (Figure 2a, blue region, and Table S1). The ABTS^+•^ solution contains residual ABTS, and may also contain traces of ABTS^2+^ formed by 2e^−^-oxidation of ABTS. The increase in ABTS^+•^ concentration caused by mepBeets can be explained by oxidation of ABTS and/or reduction of ABTS^2+^, whose absorption maxima are at 340 and 518 nm, respectively. Although the absorption spectra of the betalains and ABTS^+**•**^ do not superimpose, the spectrophotometric detection of ABTS and ABTS^2+^ is impossible due to the spectral overlap (Figure S9). 

After ca. 30 min, the TEAC of mepBeet and *m*-OH-mepBeet becomes positive due to the one-electron reduction or oxidation of ABTS^+•^ (Figure 2a). Non-phenolic and *N*-methyl betalains show lower TEAC compared to *m*-OH-pBeet, implying that both the phenolic group and the 1,7-diazaheptamethinium system are essential to explain the high radical scavenging capacity of betalains. Interestingly, the *m*-hydroxyl group had no effect on the TEAC of *N*-methyl betalains, possibly because the formation of iminoquinones is precluded due to the lack of conjugation between the hydroxyl group and the 1,7-diazaheptamethinium system. 

Electrode potentials (E*_p_*s) of pBeets and mepBeets were measured as a function of the pH to provide further insight into the effect of the structure of the betalain on its antiradical properties (Figure S10 and Figure 2b). *m*-OH-pBeet and *m*-OH-mepBeet are oxidized at an anodic potential (E*_pa_*) around 700 mV vs. Ag/AgCl, whereas the oxidation of pBeet requires a slightly higher potential (roughly E*_pa_* ~ 900 mV vs. Ag/AgCl). In both cases, the proton to electron ratio calculated from Nernst plots is one (Figure 2b), which is compatible with the oxidation of (*i*) the phenol portion to the semiquinone (–1H^+^/–1e^−^), (*ii*) the 1,7-diazaheptamethinium system to the corresponding radical cation (–1H^+^/–1e^−^), and (*iii*) the 2-piperideine ring into 2,6-dicarboxy pyridine (–2H^+^/–2e^−^) [54,55]. The phenol and 1,7-diazaheptamethinium portions of *m*-OH-pBeet and *m*-OH-mepBeet are not conjugated and, therefore, are expected to scavenge radicals independently, without the formation of iminoquinones by 2e^−^-oxidation.

### 3.3. Mechanisms of Radical Scavenging by Betalains

**Figure 3:**
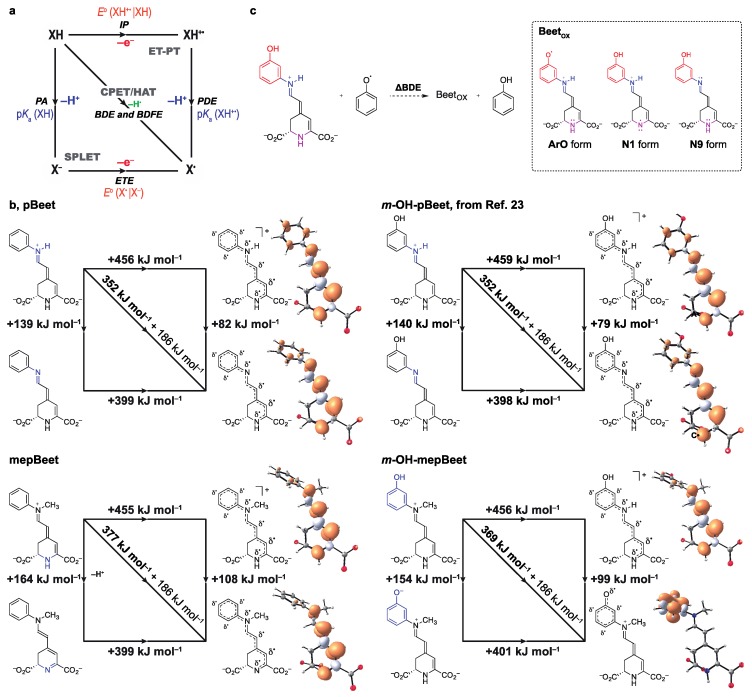
Mechanism of betanin oxidation. (**a**) More O’Ferrall–Jencks diagrams showing concurrent PCET processes of a generic compound XH [64,65], and the parameters that can be used for mechanistic interpretation, i.e. p*K*_a_s, reduction potentials (*E*^o^), proton affinity (PA), proton dissociation energy (PDE), ionization potential (IP), electron transfer enthalpy (ETE), and homolytic bond dissociation enthalpy (BDE). (**b**) More O’Ferrall–Jencks diagrams for the ionization and 1e^−^-oxidation of pBeet, *m*-OH-pBeet, mepBeet, and *m*-OH-mepBeet to produce the lowest energy radical/radical-cation; compounds are presented as dicarboxylates since this is the expected major form in water at pH higher than 4 [50,51]. Energies refer to the enthalpy changes between states. Spin density distribution (isocontour = 0.004 a.u., positive/orange and negative/white) and chemical structures show the delocalization of the unpaired electron. (**c**) Isodesmic reaction between the phenoxyl radical and betalains to produce phenol and the oxidized betalain (Beet_OX_). The designation of each betalain according to the site of deprotonation/oxidation of *m*-OH-pBeet is presented for clarity and used in Table 2.

Theoretical calculations provide in-depth insight into the mechanism of the antiradical action of betalains. Proton-coupled electron transfer (PCET) includes all processes involving electron/proton transfer [38,56]. Hydrogen atom transfer (HAT) and concerted proton–electron transfer (CPET) are examples of concerted PCET pathways [56]. Stepwise PCET occurs through sequential proton loss electron transfer (SPLET) or electron transfer followed by proton transfer (ET-PT). These concurrent reaction pathways can be represented using More O’Ferrall–Jencks (or ‘square’) diagrams and the thermodynamic preference of a given transformation can be inferred from experimental and theoretical parameters (Figure 3a). 

The homolytic bond dissociation enthalpies (BDE) of N1–*H*, N9–*H* and/or ArO–*H* bonds in all betalains are much lower than their adiabatic ionization potentials (IP) (Table 2). Consequently, the oxidation of betalains is likely to occur via either SPLET or HAT/CPET instead of by ET-PT. Kinetic and/or computational analysis is required to discriminate between HAT/CPET or SPLET mechanisms for the oxidation of betalains in water because the differences between their electron transfer enthalpies (ETE) and BDEs are lower than ca. 50 kJ mol^−1^ (12 kcal mol^−1^), independent of the site of deprotonation (Table 2) [57,58,59,60]. The lowest energy pathway for the 1H^+^/1e^−^-oxidation of each betalain is shown in Figure 3b. For *m*-OH-pBeet and pBeet, concerted PCET involving the N9–*H* moiety (N9 form, Figure 3c) is preferred over the N1–*H* (N1 form) and the phenol (ArO form), when feasible.

The occurrence of concerted PCET (HAT or CPET mechanisms) can be inferred from the degree of thermodynamic coupling between the p*K*_a_ and the *E*^o^. [38,61]. Unfortunately, the titration of betalains is difficult due to their rather limited solubility in water (≤ 10 mmol L^−1^), and fast hydrolysis and/or decomposition under very acidic or alkaline conditions [16,50,51,62,63]. Furthermore, anodic processes of pBeets and mepBeets are usually irreversible, making the calculation of formal redox potentials difficult (Figure S10). Therefore, to determine whether the oxidation of betalains occurs through HAT/CPET or SPLET, we calculated the change in the BDE using the isodesmic reaction between phenoxyl radical/phenol (PhO^•^/PhOH) and betalains (Figure 3c). Changes in enthalpy for the reaction of either pBeet or *m*-OH-pBeet with the phenoxyl radical are negative when the hydrogen atom attached to N9 is transferred. For *N*-methyl betalains, however, the process is endothermic, independent of the deprotonation site, i.e., N1–*H* or ArO–*H* (Table 2). From these results, we infer that changes in the acidity of N1–*H* and N9–*H* and the *E*^o^ are strongly related, indicating that HAT/CPET is the preferred thermodynamic pathway for the 1e^−^-oxidation of betalains. 

The analysis of the spin density distribution of the lowest energy 1H^+^/1e^−^-oxidized betalain revealed that the phenoxyl radical of *m*-OH-mepBeet is not delocalized over the 1,7-diazaheptamethinium system (Figure 2b). Conversely, for mepBeet, the spin density is almost fully located on the 1,7-diazaheptamethinium system after 1e^−^-oxidation and loss of the proton attached to N1. These results confirm the importance of both moieties for the overall radical scavenging capacity of betalains and indicate that they do not have to be conjugated. For betanin, the 5-*O*-glucosylated hydroxyl group of the cyclo-DOPA ((*S*)-2-carboxy-5,6-dihydroxyindoline) moiety is conjugated to the 1,7-diazaheptamethinium portion, but the 6-OH group is not, pointing to multiple sites of oxidation and supporting the hypothesis of PCET-mediated hole stabilization by betanin in nano-hybrid plasmonic systems for hydrogen production [19].

## 4. Conclusions

*m*-OH-pBeet, a model compound for the antioxidant action of betalains, has three potential sites for PCET: the phenol moiety, the 1,7-diazaheptamethinium system and the 2-piperideine ring. For *m*-OH-mepBeet, the presence of a methyl group attached to the N9 makes the oxidation of the 1,7-diazaheptamethinium system less favorable, lowers its radical scavenging capacity compared to *m*-OH-pBeet and promotes pro-oxidant action under acidic conditions. The deprotonation of the N1-*H* is the only pathway for the oxidation of the 1,7-diazaheptamethinium system of *m*-OH-mepBeet, but the resulting radical ion is not susceptible to further oxidation. Since pBeet performs much better than *m*-OH-mepBeet at near neutral conditions, the 1,7-diazaheptamethinium system is clearly more important for the overall radical scavenging capacity of betalains than the phenolic *m*-hydroxyl moiety. Concerted PCET involving the proton at N9–*H* of the 1,7-diazaheptamethinium system is the thermodynamically most favorable mechanism for the oxidation of *m*-OH-pBeet and pBeet, while for mepBeet and m-OH-mepBeet the preferred pathways are the less spontaneous oxidation of the 1,7-diazaheptamethinium system via the N1 proton and the phenol, respectively. The present results thus demonstrate that the 1,7-diazaheptamethinium moiety is the key structural feature responsible for the efficient concerted PCET radical scavenging capacity of betalains even in the absence of conjugation to a phenol moiety.

## Figures and Tables

**Table 1:** Absorption and fluorescence properties of pBeets and mepBeets. ^a^

Compound	λ^abs^ (nm)	ε ^b^	λ^EM^ (nm)	Δλ (cm^−1^)	Φ_Fl_ (/ 10^−3^) ^c^	E_S_ (kJ mol^−1^)
pBeet	508	61,300	558	1760	1.29 ± 0.04	220
mepBeet	492	60,600	553	2240	0.56 ± 0.04	230
*m*-OH-pBeet	508	64,000	563	1880	0.85 ± 0.03	220
*m*-OH-mepBeet	492	60,500	550	2140	0.39 ± 0.02	230

^a^ In Britton–Robinson buffer (pH 6, 40 mmol L^−1^); ^b^ In L mol^−1^ cm^−1^, measured at pH 7 (pBeets) or 11 (mepBeets). ^c^ Using rhodamine B in ethanol as standard (*n*_D_ = 1.3616; Φ_Fl_ = 0.5) [52].

**Table 2:** Thermodynamic parameters of pBeets and mepBeets for the three most common radical scavenging mechanisms. ^a.^

Species ^b^	ET-PT		HAT/PCET	SPLET		PhO^•^→PhOH
	IP	PDE	BDE	PA	ETE	ΔBDE^PhOH^
pBeet	456					
N9		82	352	139	399	–11
N1		110	380	161	405	17
mepBeet	455					
N1		108	377	164	399	14
*m*-OH-pBeet ^c^	459					
N9		79	352	140	398	–12
ArO		96	369	154	401	5
N1		109	382	162	406	18
*m*-OH-mepBeet	456					
ArO		99	369	154	401	6
N1		108	378	164	400	15

^a^ Values (in kJ mol^−1^) were calculated using Equations (1)–(6), see Methods. ^b^ Species refer to the parent species and the deprotonation site (Figure 3c). ^c^ from Reference [27].

## Supplementary Materials

The following are available online at https://www.mdpi.com/2076-3921/8/7/222/s1, Supplementary Methods, Figure S1: ^1^H NMR spectrum (500 MHz, CD_3_OD) of pBeet, Figure S2: ESI(+)-MS spectrum of pBeet, Figure S3: ^1^H NMR spectrum (500 MHz, CD_3_OD) of mepBeet, Figure S4: ESI(+)-MS spectrum of mepBeet, Figure S5: ^1^H NMR spectrum (500 MHz, CD_3_OD) of *m*-OH-pBeet, Figure S6: ESI(+)-MS spectrum of *m*-OH-pBeet, Figure S7: ^1^H NMR spectrum (500 MHz, CD_3_OD) of *m*-OH-mepBeet, Figure S8: ESI(+)-MS spectrum of *m*-OH-mepBeet, Figure S9: Normalized absorption spectra of pBeets, mepBeets and ABTS^+•^, Figure S10: Cyclic voltammograms of pBeets and mepBeets in BR buffer at pH ranging from 3 to 7, Figure S11: UV-Vis spectra of pBeet, mepBeet, *m*-OH-pBeet and *m*-OH-mepBeet over time, and Table S1: TEAC values ± sd of the pBeets and the mepBeets.
