# Supplementary material for: Rationale on the High Radical Scavenging Capacity of Betalains
Source: Antioxidants (Basel). 2019 Jul 13;8(7):222. doi: 10.3390/antiox8070222 (PMC6680464; doi:10.3390/antiox8070222)
Supplement: Supplementary file 1 [file antioxidants-08-00222-s001.pdf]

SUPPLEMENTARY MATERIALS

Rationale on the high radical scavenging capacity of betalains

Karina K. Nakashima and Erick L. Bastos

Summary

Supplementary methods S3

Solutions and buffers S3

Molar absorption coefficient (ε) S3

Fluorescence quantum yields (Φ_{FL}) S3

Cyclic Voltammetry S4

NMR spectroscopy S4

HPLC-DAD-ESI(+)-MS/MS analyses S5

Supplementary Figures and Table S6

Figure S1. ^{1}H NMR spectrum (500 MHz, CD_3OD) of pBeet. S6

Figure S2. ESI(+)-MS spectrum of pBeet. S6

Figure S3. ^{1}H NMR spectrum (500 MHz, CD_3OD) of mepBeet. S7

Figure S4. ESI(+)-MS spectrum of mepBeet. S7

Figure S5. ^{1}H NMR spectrum (500 MHz, CD_3OD) of *m*-OH-pBeet. S8

Figure S6. ESI(+)-MS spectrum of *m*-OH-pBeet. S8

Figure S7. ^{1}H NMR spectrum (500 MHz, CD_3OD) of *m*-OH-mepBeet. S9

Figure S8. ESI(+)-MS spectrum of *m*-OH-mepBeet S9

Figure S9. Normalized absorption spectra of pBeets, mepBeets and $ABTS^{+•}$ S10

Figure S10. Cyclic voltammograms of pBeets and mepBeets in BR buffer at pH ranging from 3 to 7. .... S10

Figure S11. Hydrolysis of pBeet, mepBeet, *m*-OH-pBeet and *m*-OH-mepBeet. S11

Table S1. TEAC values ± sd of the pBeets and the mepBeets. S12

References S13

Supplementary methods

Solutions and buffers

Britton-Robinson (BR) universal buffers[1] (40 mmol L^{-1}, pH 3 - 7) were prepared by mixing aqueous solutions of H_3BO_3 (40 mmol L^{-1}), H_3PO_4 (40 mmol L^{-1}), and H_3CCO_2H (40 mmol L^{-1}) and adjusting the pH to the desired value with aqueous NaOH solution (0.2 mol L^{-1}) at 25 ºC.

Molar absorption coefficient (ε)

Since the direct weighting of betalains is difficult due to limited amount of substance and high hygroscopicity, the molar absorption coefficient (ε) of the pBeets and the mepBeets was determined using an endpoint method[2] (Figure S11). Briefly, the alkaline hydrolysis of betalains in BR buffer (pH 7 or 11, 40 mmol L^{-1}) was spectrophotometrically monitored. The initial pigment concentration and ε of all betalains were determined by comparing the absorption of the resulting betalamic acid solution ($\varepsilon^{424\,nm}$ = 27,000 L mol^{-1} cm^{-1})[3] with that of betanin solutions of known concentrations. The betalamic acid solutions were stable under the experimental conditions, and no appreciable change in the spectral properties could be detected after 30 min.

Fluorescence quantum yields (Φ_{FL})

Fluorescence quantum yields (Φ_{FL}) were determined using rhodamine B as the secondary fluorescence standard (ethanol solution; n_D = 1.3616; Φ_{Fl} = 0.5)[4]. Fluorescence spectra of the standard and the betalains were taken under identical spectrometer conditions (excitation (EX) at 490 nm, emission (EM) wavelength range: 510 – 800 nm, slits 20 nm (EX and EM), photomultiplier power 600 V, 25 ± 1 ºC) on a Varian Eclipse spectrofluorimeter equipped

with a thermostatized cell holder. Plots of the integrated fluorescence intensity *vs.* absorbance were submitted to linear regression analysis and the slopes α_X and α_S were used to calculate the Φ_{FL} using Eq. S1.

$$\Phi_{Fl}^{X} = \Phi_{FL}^{S}\left(\frac{\alpha_X}{\alpha_S}\right)\left(\frac{n_X}{n_S}\right)^2 \qquad \text{(Eq. S1)}$$

where n is the refractive index of the solvent and Φ_{FL} are the absolute quantum yields; X refers to the sample and S to the standard.

Cyclic Voltammetry

Measurements were carried out in a Autolab PGSTAT101 potentiostat/galvanostat controlled with NOVA software and equipped with a 10 mL conventional electrochemical cell at room temperature. A glassy carbon working electrode (diameter of electrode disk = 2 mm), a platinum wire auxiliary electrode and and a Ag|AgCl (KCl, sat.) reference electrode were used; scan rate: 50 mV s^{-1}, potential range: –1.0 to 1.0 V or –1.0 to 1.25 V; [betalain] = 0.1 mmol L^{-1} (in BR buffer). Before each experiment, the working electrode was polished with 0.05 µm sized aluminum oxide particles (60 cycles) and washed with water under ultrasound irradiation for 1 min. The number of proton lost/gained by electron ratio (v/n) was calculated from the Nernst plot (Figure 2); Nernst limit of –59 mV/pH (v/n = 1).

NMR spectroscopy

The ^{1}H NMR spectra of betalains were recorded using a Bruker Avance III 500 (11.7 T) spectrometer, operating at 500 MHz, using a 5-mm triple resonance probe (TXI) with inverse detection, at 293 K, and the *zg30* pulse program (30º flip angle). Baseline correction was performed using the Whittaker smoother algorithm as implemented in the MestReNova software (v.8.1.4, 2013, Mestrelab Res.). Samples were prepared in CD_3OD (700 µL)

immediately before spectral acquisition at a concentration of 4.0 mg mL^{-1}. Chemical shifts are reported as δ values (ppm) referenced to sodium trimethylsilyl propionate-d_4 (TSP) signal, δ_H (TSP, *s*) = 0 ppm. Multiplicities were given as *s* (singlet); *brs* (broad singlet); *d* (doublet); *t* (triplet); *m* (multiplet) and, *dd* (double of doublets). Due to limited solubility and low persistence of these betalains in D_2O and in other polar solvents, ^{13}C NMR data could not be obtained.

HPLC-DAD-ESI(+)-MS/MS analyses

Experiments were carried out on an Bruker Daltonics Esquire HCT ion trap mass spectrometer equipped with an electrospray source and coupled to a Shimadzu Prominence LC-20AD liquid chromatograph equipped with a Kinetex EVO C18 column (150 mm × 4.6 mm, 5 µm, Phenomenex) maintained at 25 ºC, and a PDA SPD-M20A detector. Nitrogen was used as nebulizing (45 psi) and drying gas (6 L min^{-1}, 325 ºC) and helium as buffer gas (4×10^{-6} mbar). The capillary high voltage was set to 4 kV. To avoid space-charge effects, smart ion charge control (ICC) was set to the arbitrary value of 50,000. LC analysis conditions: linear gradient from 5% to 20% B in 8 min and 20 to 50% B from 8 to 10 min, at 25 ºC; solvent systems, A: water with formic acid (0.05% v/v); B: 0.05% v/v formic acid in MeCN/water (60/40); flow rate: 0.3 mL min^{-1}, injection volume: 10 µL.

Supplementary Figures and Table

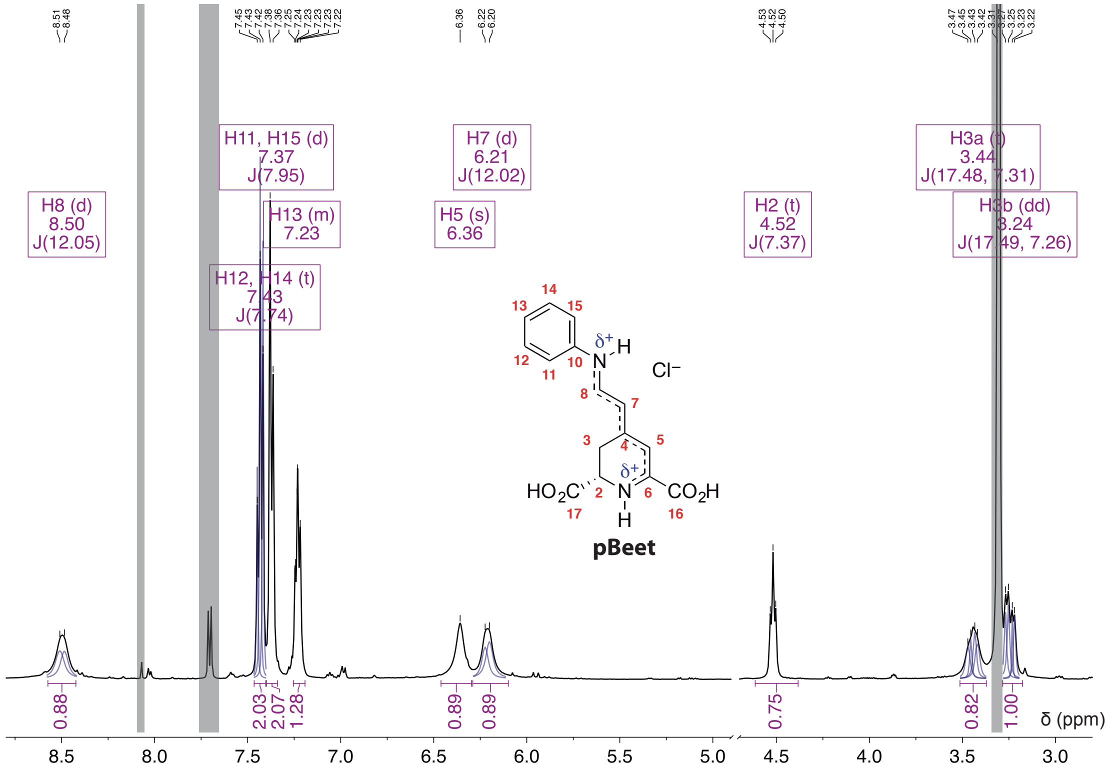

Figure S1. ¹H NMR spectrum (500 MHz, CD_3OD) of pBeet. The interval between 4.6 – 4.8 ppm (residual water signal) is not shown for clarity. The multiplicity of poorly-resolved peaks were inferred by using non-linear data fitting (blue curves).

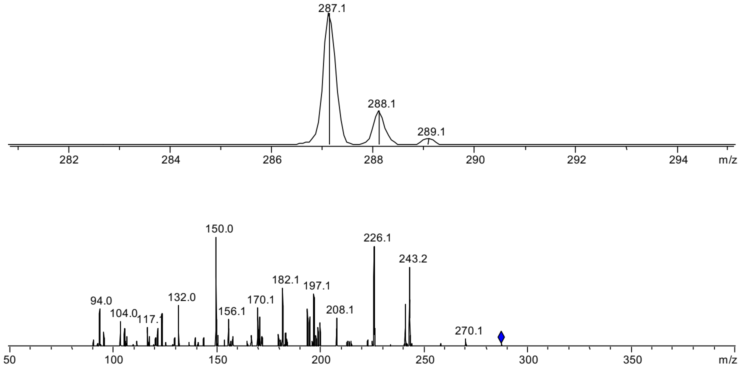

Figure S2. ESI(+)-MS spectrum of pBeet (exact mass: 287.1026 amu) and MS/MS spectrum of the precursor ion *m/z* 287.1.

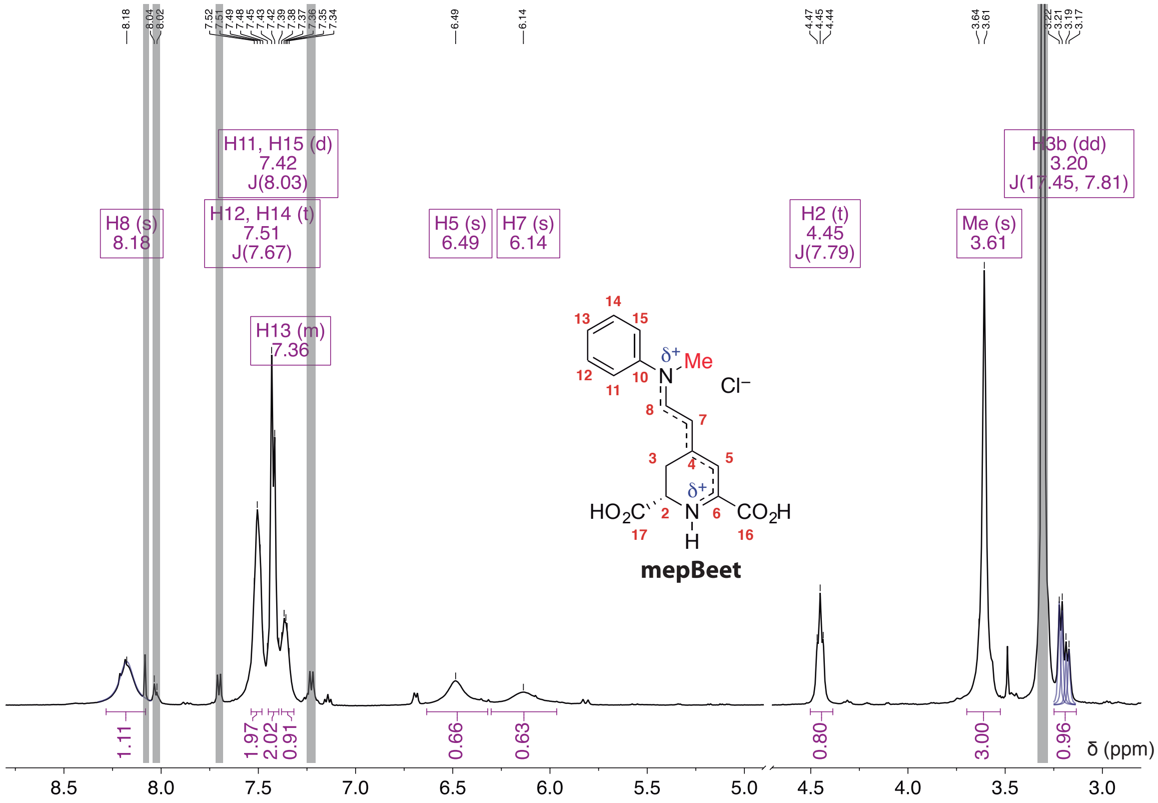

Figure S3. ^{1}H NMR spectrum (500 MHz, CD_3OD) of mepBeet. The interval between 4.6 – 4.8 ppm (residual water signal) is not shown for clarity. The multiplicity of poorly-resolved peaks were inferred by using non-linear data fitting (blue curves).

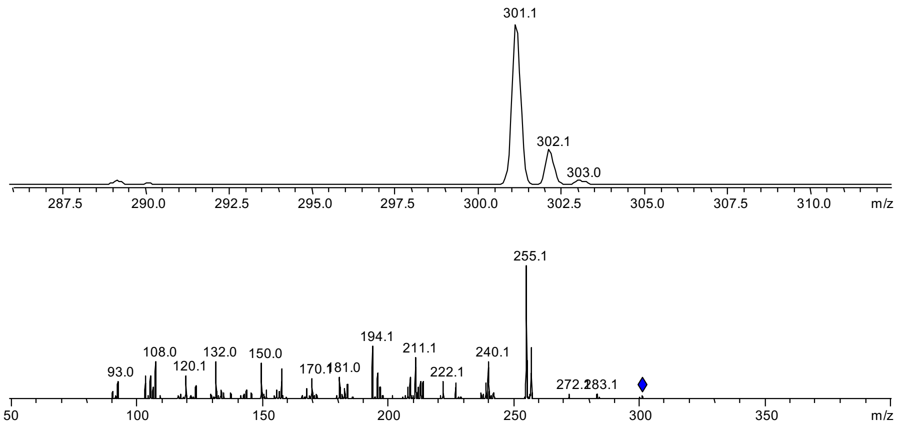

Figure S4. ESI(+)-MS spectrum of mepBeet (exact mass: 301.1183 amu) and MS/MS spectrum of the precursor ion *m/z* 301.1.

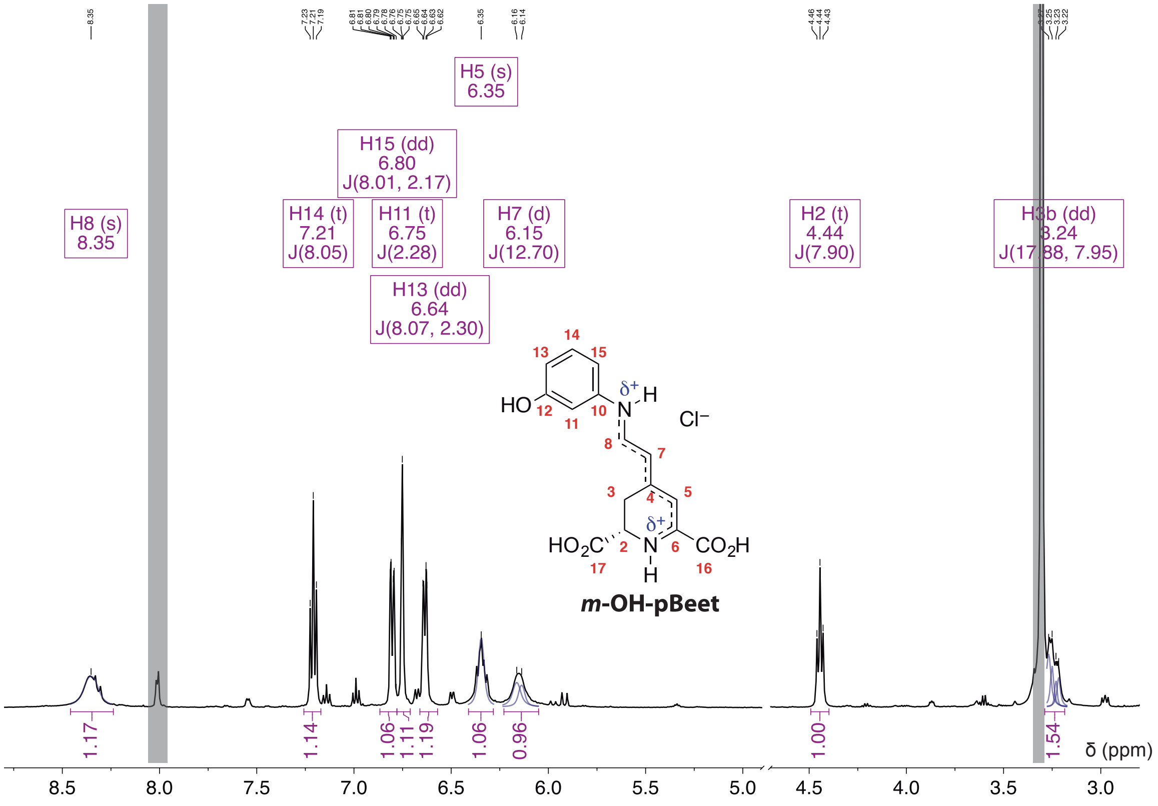

Figure S5. ^{1}H NMR spectrum (500 MHz, CD_3OD) of *m*-OH-pBeet. The interval between 4.6 – 4.8 ppm (residual water signal) is not shown for clarity. The multiplicity of poorly-resolved peaks were inferred by using non-linear data fitting (blue curves).

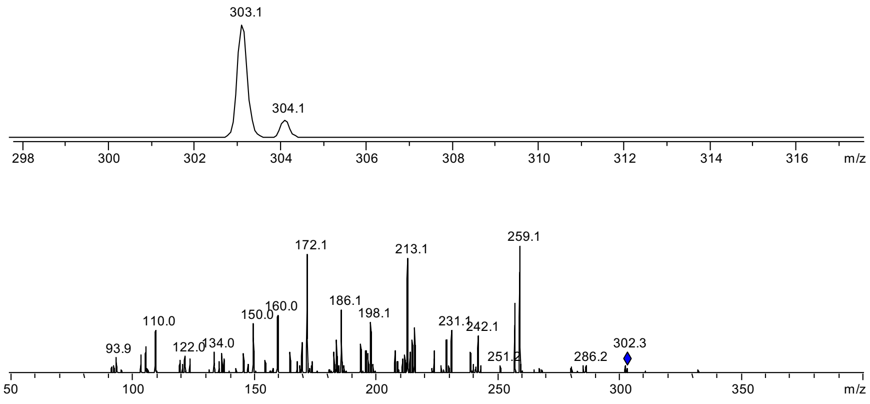

Figure S6. ESI(+)-MS spectrum of *m*-OH-pBeet (exact mass: 303.0975 amu) and MS/MS spectrum of the precursor ion *m*/*z* 303.1.

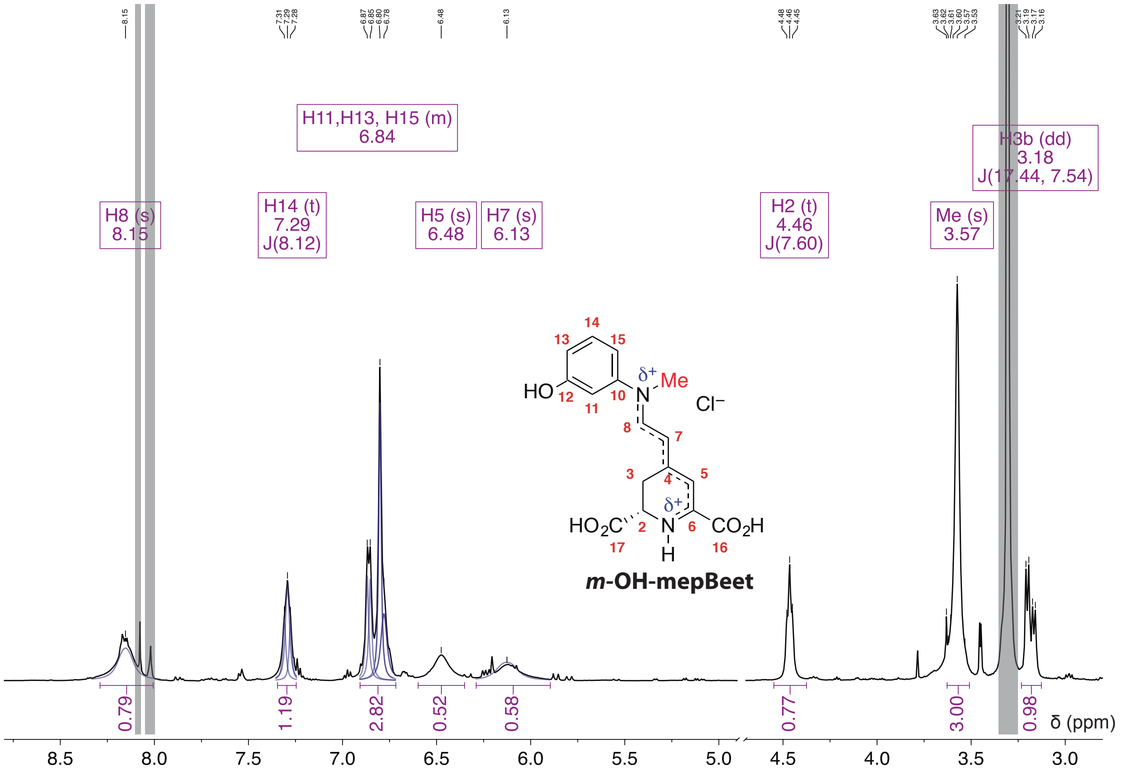

Figure S7. ^{1}H NMR spectrum (500 MHz, CD_3OD) of *m*-OH-mepBeet. The interval between 4.6 – 4.8 ppm (residual water signal) is not shown for clarity. The multiplicity of poorly-resolved peaks were inferred by using non-linear data fitting (blue curves).

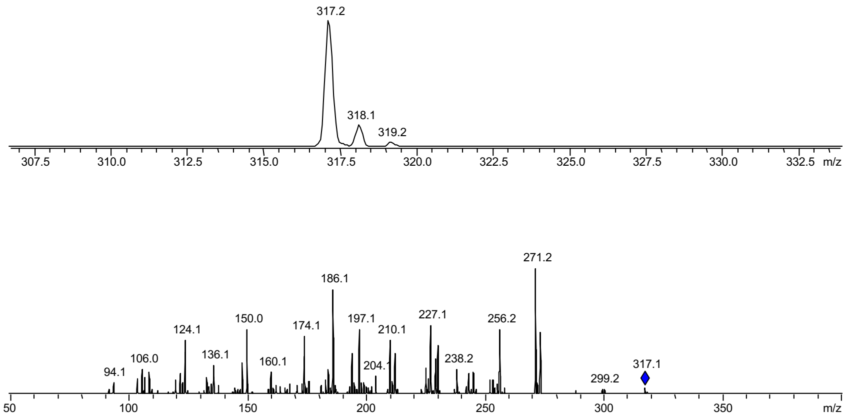

Figure S8. ESI(+)-MS spectrum of *m*-OH-mepBeet (exact mass: 317.1132 amu) and MS/MS spectrum of the precursor ion *m*/*z* 317.2

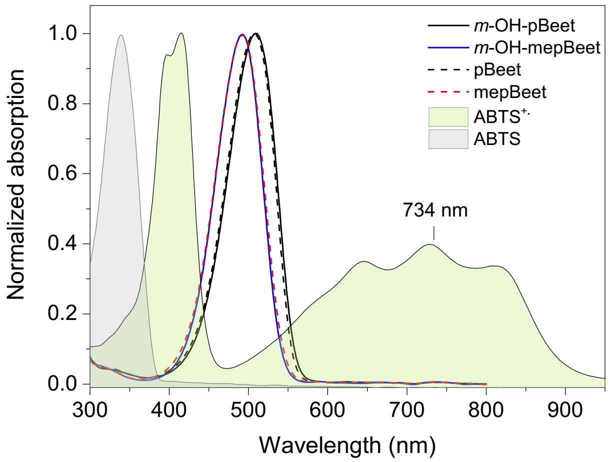

Figure S9. Normalized absorption spectra of pBeets, mepBeets and $ABTS^{+•}$ in water.

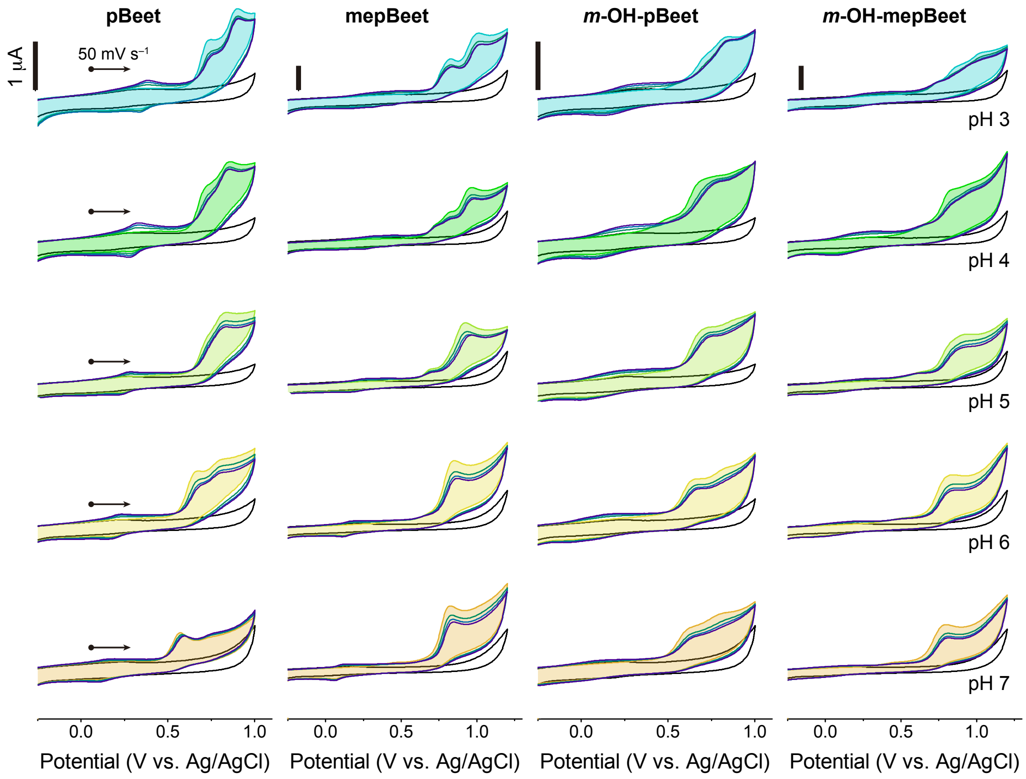

Figure S10. Cyclic voltammograms of pBeets and mepBeets in BR buffer at pH ranging from 3 to 7. Glassy carbon electrode; Ag|AgCl (KCl sat.); scan rate: 50 mV s^{-1}, potential range: –1.0 to 1.0 V or –1.0 to 1.25 V; [betalain] = 0.1 mmol L^{-1}.

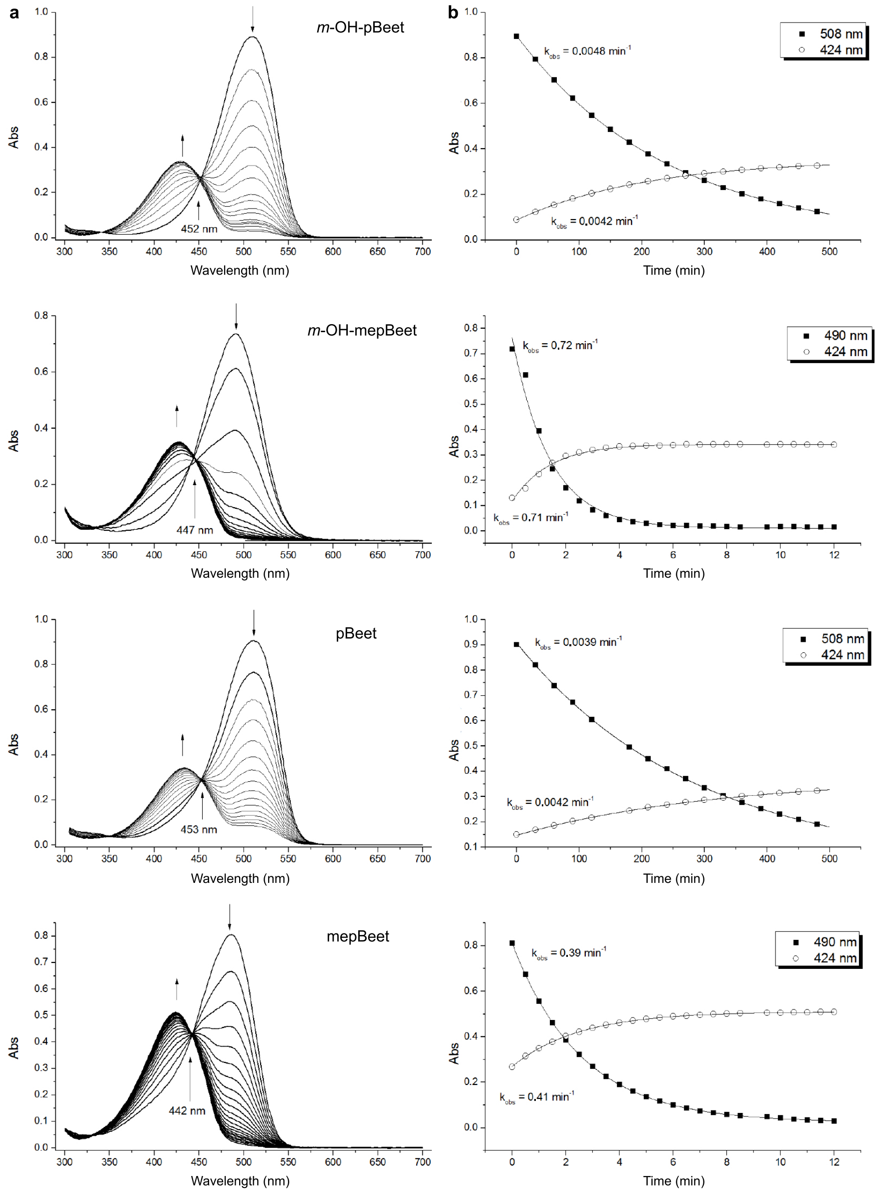

Figure S11. Hydrolysis of pBeet, mepBeet, *m*-OH-pBeet and *m*-OH-mepBeet. (a) Absorption spectra of betalains in BR buffer (pH 7 or 11, 40 mmol L^{-1}). (b) Kinetics of betalain hydrolysis and betalamic acid formation, as inferred from changes in absorption at roughly 500 nm and 424 nm, respectively. The observed rate constants (k_{obs}) were calculated by non-linear curve fitting of the experimental data to a monoexponential function.

Table S1. TEAC values ± sd of the pBeets and the mepBeets measured after 6 – 120 min of reaction.

pH	*m*-OH-pBeet	*m*-OH-mepBeet	pBeet	mepBeet
**6 min**				
**3**	2.5 ± 0.2	−0.9 ± 0.1	1.5 ± 0.1	−0.9 ± 0.1
**4**	2.0 ± 0.1	−1.4 ± 0.1	1.5 ± 0.1	−0.8 ± 0.1
**5**	2.6 ± 0.2	−0.4 ± 0.1	3.1 ± 0.2	−0.4 ± 0.1
**6**	4.0 ± 0.2	0.5 ± 0.1	2.7 ± 0.2	0.3 ± 0.1
**7**	3.8 ± 0.2	1.1 ± 0.1	2.0 ± 0.1	1.2 ± 0.1
**30 min**				
**3**	3.6 ± 0.3	1.2 ± 0.2	2.3 ± 0.1	−0.2 ± 0.1
**4**	3.5 ± 0.1	0.5 ± 0.7	2.0 ± 0.3	0.6 ± 0.1
**5**	4.2 ± 0.2	1.4 ± 0.1	3.7 ± 0.1	1.0 ± 0.1
**6**	5.5 ± 0.2	1.4 ± 0.1	3.9 ± 0.2	2.3 ± 0.1
**7**	5.4 ± 0.2	1.7 ± 0.1	3.0 ± 0.1	2.2 ± 0.1
**60 min**				
**3**	3.6 ± 0.2	2.3 ± 0.2	2.4 ± 0.1	0.9 ± 0.1
**4**	4.3 ± 0.2	1.3 ± 0.2	2.6 ± 0.2	1.5 ± 0.1
**5**	4.8 ± 0.2	1.8 ± 0.1	4.0 ± 0.2	1.9 ± 0.1
**6**	5.3 ± 0.2	1.7 ± 0.1	3.8 ± 0.2	3.0 ± 0.1
**7**	5.6 ± 0.2	1.8 ± 0.1	3.2 ± 0.1	2.4 ± 0.1
**120 min**				
**3**	4.3 ± 0.2	2.3 ± 0.2	2.5 ± 0.1	1.7 ± 0.2
**4**	4.8 ± 0.2	1.8 ± 0.2	2.7 ± 0.1	2.1 ± 0.1
**5**	4.8 ± 0.2	1.9 ± 0.1	3.7 ± 0.2	2.2 ± 0.1
**6**	5.2 ± 0.2	1.9 ± 0.1	3.5 ± 0.1	3.2 ± 0.1
**7**	5.1 ± 0.2	2.0 ± 0.1	3.1 ± 0.2	2.7 ± 0.1

References

1. Britton, H.T.S.; Robinson, R.A. Universal buffer solutions and the dissociation constant of veronal. *J. Chem. Soc. Res.* **1931**, 1456-1462.
2. Gandia-Herrero, F.; Escribano, J.; Garcia-Carmona, F. Structural implications on color, fluorescence, and antiradical activity in betalains. *Planta* **2010**, *232*, 449-460, doi:10.1007/s00425-010-1191-0.
3. Gandia-Herrero, F.; Escribano, J.; Garcia-Carmona, F. Purification and antiradical properties of the structural unit of betalains. *J. Nat. Prod.* **2012**, *75*, 1030-1036, doi:10.1021/np200950n.
4. Karstens, T.; Kobs, K. Rhodamine-B and rhodamine-101 as reference substances for fluorescence quantum yield measurements. *J. Phys. Chem.* **1980**, *84*, 1871-1872, doi:DOI 10.1021/j100451a030.
